# Association between a history of periodontitis and the risk of systemic lupus erythematosus in Taiwan: A nationwide, population-based, case-control study

**DOI:** 10.1371/journal.pone.0187075

**Published:** 2017-10-23

**Authors:** Yi-Da Wu, Ching-Heng Lin, Wen-Cheng Chao, Tsai-Ling Liao, Der-Yuan Chen, Hsin-Hua Chen

**Affiliations:** 1 Division of Allergy, Immunology and Rheumatology, Department of Internal Medicine, Taichung Veterans General Hospital, Taichung, Taiwan; 2 Department of Medical Research, Taichung Veterans General Hospital, Taichung, Taiwan; 3 Department of Healthcare Management, National Taipei University of Nursing and Health Sciences, Taipei, Taiwan; 4 Division of Chest Medicine, Department of Internal Medicine, Taichung Veterans General Hospital, Taichung, Taiwan; 5 Department of Business Administration, National Changhua University of Education; 6 Institute of Biomedical Science and Rong Hsing Research Center for Translational Medicine, Chung-Hsing University, Taichung, Taiwan; 7 School of Medicine, National Yang-Ming University, Taipei, Taiwan; 8 School of Medicine, Chung-Shan Medical University, Taichung, Taiwan; 9 Department of Medical Education, Taichung Veterans General Hospital, Taichung, Taiwan; 10 Institute of Public Health and Community Medicine Research Center, National Yang-Ming University, Taipei, Taiwan; Keio University, JAPAN

## Abstract

**Objective:**

To examine the association between a history of periodontitis (PD) and incident systemic lupus erythematosus (SLE)

**Methods:**

We used 2003–2012 claims data from the Taiwanese National Health Insurance Database to identify 7,204 incident SLE patients during 2007–2012 as the study group, along with randomly selecting 72,040 non-SLE patients matched (1:10) for age, gender, and first diagnosis date (index date) as the control group. The correlation between PD and SLE risk was estimated using conditional logistic regression analysis, after making adjustments for confounders (including a history of diabetes and number of non-PD related dental visits before the index date). To evaluate the effects of PD severity and the lag time which occurred since the last PD visit on SLE development, odds ratios (ORs) with 95% confidence intervals (CIs) were calculated for subgroups of patients with PD according to their number of visits, cumulative cost and also the time gaps between their last PD-related visit and the index date.

**Results:**

A statistically significant association between a history of PD and newly diagnosed SLE was observed (OR, 1.21; 95% CI, 1.14–1.28; p-value, <0.001). The association was both dose- and time-dependent and was found to be strongest when the interval between the last PD-related visit and the index date was less than three months (OR, 1.83; 95% CI, 1.61–2.09; p-value, <0.001). The association between PD exposure and SLE risk was consistently significant among subgroups stratified based on age, gender, or DM status.

**Conclusions:**

The results of this nationwide, population-based, case-control study suggest that there is a significant association between a history of PD and incident SLE in Taiwan. This weak association is limited to lack of information on individual smoking status in the database.

## Introduction

Systemic lupus erythematosus (SLE) is a systemic autoimmune disease affecting various organs while having a broad spectrum of clinical presentation results stemming from chronic and recurrent activation of the immune system. Due to the improved detection ability found in milder cases, the incidence of SLE has increased over the last 40 years [1]. The reported prevalence of SLE ranges from 20 to 240 cases per 100,000 persons, while the incidence rates range from 1 to 10 per 100,000 person-years [2]. The etiology of SLE is multifactorial. Many observations have suggested a role for genetic, hormonal, immunologic, and environmental factors. Among these, infections may play a pivotal role in the expression of the disease in a genetically susceptible individual [3, 4]. Infection agents have been hypothesized as acting as triggers for autoimmune disease through molecular mimicry, alterations in self-antigens, immune cell activation or infection-mediated inflammation [5–7]. Epidemiologic studies have demonstrated a higher prevalence of antibodies specific for the Epstein-Barr Virus (EBV) antigen in children with SLE when compared with general population. They have also shown a higher frequency of anti-EBV antibodies in a military cohort before the diagnosis of lupus, supporting a possible role for that virus in disease pathogenesis [8]. Additionally, mycobacterial or trypanosomiasis infections may induce anti-DNA antibodies or even lupus-like symptoms, while the occurrence of lupus flares may follow bacterial infections [9, 10]. A nationwide, population-based study in Taiwan has suggested that prior TB infection may play a role in precipitating SLE [11].

Periodontitis (PD) is characterized by chronic inflammation caused by bacterial infections present in the dental biofilm [12] and may predispose itself towards the development of particular diseases. Many studies have shown a potential association between chronic PD and autoimmune diseases [13], particularly rheumatoid arthritis (RA). Our previous nationwide, population-based study revealed that there was an association between a history of PD and RA risk and that such an association was both dose- and time-dependent [14]. Nesse et al. reported that the prevalence of cardiovascular and autoimmune diseases increased in PD patients [15]. PD exhibits very similar characteristics to SLE pathophysiology. A large number of B cells and plasma cells have also been detected in periodontal lesions [16]. A high prevalence of PD had been reported in SLE patients [17, 18]. Kobayashi et al. and Chai et al. found that Fcγ receptor polymorphisms were associated with PD and other autoimmune diseases, such as RA and SLE [17, 19]. In a Japanese population, the combination of stimulatory FcγRIIA and inhibitory FcγRIIB genotypes may increase susceptibility to both SLE and PD [20]. Wang et al. reported that elevated anti-cardiolipin and anti-β2-glycoprotein I antibody levels were associated with periodontal bacteria and periodontal breakdown in SLE patients [21]. Novo E et al. showed that there was a high number of ANCA-positive sera in SLE patients, particularly in those with PD [18]. Furthermore, Fabbri C et al. reported that PD treatment improved response to immunosuppressive therapy in SLE patients [22]. To date, there remains limited real-world data regarding the association between PD and the risk of SLE development. The Taiwanese National Health Insurance Research Database (NHIRD) has released claims data to facilitate population-based longitudinal studies. The purpose of this study was to investigate the association between a history of PD and newly diagnosed SLE through the use of the NHIRD.

## Methods

### Ethics statement

This study was approved by the ethics committee for clinical research at Taichung Veterans General Hospital (IRB TCVGH No: CE16251A). Informed consent was not acquired since all personal information that could be traced was anonymised before analysis of data.

### Study design

The study used a retrospective, case-control study design.

### Data source

The National Health Insurance (NHI) Program has been implemented in Taiwan since 1995 and covers more than 98% of the population in Taiwan. It includes data on ambulatory care, inpatient services, dental services, and prescription drugs. The NHIRD is managed by the National Health Research Institute and is comprised of comprehensive NHI-related administrative and claims data for research purposes. This study utilized multiple NHIRD datasets, including NHI catastrophic illness files, 2003–2012 ambulatory and inpatient claims files, along with enrollment files.

The NHI registry system for catastrophic illnesses tracks patients with major or catastrophic illnesses, including some autoimmune diseases such as SLE, RA and Sjögren’s syndrome (SS). Patients with a catastrophic illness certificate are exempt from providing any copayment. The Bureau of NHI (BNHI) performs a routine validation of diagnosis, which is handled by at least two specialists who carefully review original medical records, laboratory data, imaging findings and the pathological results of all patients who seek to be included in catastrophic illness registration. The BNHI only issues catastrophic illness certificates to those who meet the classification criteria for major illness. NHI catastrophic illness files include ambulatory and inpatient claims of beneficiaries and are distributed as a package. The 2003–2012 ambulatory and inpatient files provide information on diagnosis, prescription, date of visit/admission and medical expenses. Enrollment files contain demographic and enrollment information. In 2000, the NHIRD also established a representative database taken from the entire set of enrollees by randomly selecting 1,000,000 subjects in 2000. All enrollment and utilization information associated with this random sample is available.

Although laboratory and radiographic data are not available in the database, the BNHI routinely audits the accuracy of diagnoses through the random sampling of patient charts. This BNHI auditing system has enhanced coding accuracy.

### Study samples

We identified patients who were newly diagnosed with SLE (International Classification of Disease Diagnoses, ninth revision [ICD-9-CM] code 710.0) from outpatient claim files or hospitalization records during the period 2007–2012. The American College of Rheumatology classification criteria for SLE (1997) was used for SLE diagnosis [23]. Only patients with a catastrophic illness certificate were included in our study. A catastrophic illness certificate for SLE was approved after at least two qualified rheumatologists had validated the diagnosis of SLE upon a thorough review of medical records, a lupus laboratory test, and patients’ prescription medication history. Patients diagnosed before 1 January 2007 were excluded. The index date of the study group was identified as the time of the first ambulatory care visit which resulted in an SLE diagnosis between 2007 and 2012. Due to the intrinsically increased prevalence of PD in patients with SS or RA [24–26], we excluded those who had SS or RA from our study.

The controls were randomly selected from individuals in the database without SLE, at a ratio of 1:10 (patients vs. controls). In the control group, the time of the first ambulatory visit during the matched index year was selected as the index date.

### Definition of PD

In Taiwan, people are encouraged by the BNHI to receive regular dental check-ups. Dentists may perform scaling for individuals who have subclinical periodontitis (ICD9-CM codes 523.3–523.5). In this study, patients who had one or more outpatiet visit before the index date which diagnosed them as having PD (ICD9-CM codes 523.3–523.5), and who were concurrently treated with antibiotics, or dental scaling ≥ 3 times per year by certified dentists, were identified as patients with a history of PD [14].

### Surrogate measures of PD severity

The cumulative number of PD-related visits and their cumulative costs were divided into equal quartiles, Q1–Q4, representing an increase in both visit times and PD-related costs. We assume that these measurements were positively related to the severity of periodontal disease. The PD-related visits cumulative costs were calculated by summing up all the expenses related to outpatient visits with ICD-9-CM codes 523.3–523.5, before the index date. The cost was converted from new Taiwan Dollars (TW$) to US dollars (US$), using a conversion rate of 30 TW$ to 1 US$.

### Potential confounders

Potential confounders included both type 1 and type 2 diabetes mellitus (DM). The previous study revealed DM to be an important risk factor for PD (odds ratio [OR] = 3) [27, 28]. Patients who had had at least one ambulatory visit with ICD-9-CM codes 250.x and a concurrent prescription of antidiabetic drugs were identified as patients with DM. Although this case-control study compared the odds of PD before the index date between SLE cases and controls, we cannot exclude the possibility that SLE related dental disorder, such as dental caries due to dry mouth, may occur before SLE diagnosis, leading to a higher chance to diagnose PD in SLE cases compared with controls. Therefore, we also adjusted the cumulative number of non-PD related dental visits (excluding ICD-9-CM code 523.x) in the multivariable conditional logistic regression analyses to minimize detection bias. We also transformed the cumulative number of non-PD related dental visits into a categorical variable based on each quartile.

### Statistical analysis

We used a chi-square test to compare the baseline characteristics between cases and controls for categorical variables and conditional logistic regression analysis to examine the correlations between PD and the risk of SLE, as shown by ORs with 95% Confidence Intervals (CIs). We examined the significance of the interaction between each covariate (age group, sex and DM) and PD on the risk of SLE using the Wald test to estimate the p-value of the coefficient associated with the product of each indicator of the covariate and the indicator of PD. A two-tailed p-value of <0.05 was considered statistically significant. We conducted all statistical analyses using SAS statistical software, version 9.2 (SAS Institute, Cary, NC).

## Results

Table 1 revealed baseline demographic data of the two groups. A total of 7,204 cases of SLE for those with a certificate of catastrophic illness, and 72,040 matched non-SLE controls selected from the 1,000,000 control subjects were included in the data analysis (Table 1). The proportions of pre-existing DM were not significantly different between the two groups.

**Table 1:** Comparison of demographic data, a history of periodontitis and other pre-existing diseases between patients with SLE and patients without SLE.

	Non-SLE patients	SLE patients	P-value
	(*n* = 72,040)	(*n* = 7,204)	
**Age, years** (mean ± SD)	40 ± 18	40 ± 18	1.000
<50 years	51,460 (71.4)	5,146 (71.4)	
≥50 years	20,580 (28.6)	2,058 (28.6)	
**Sex**			1.000
Female	61,990 (86.0)	6,199 (86.0)	
Male	10,050 (14.0)	1,005 (14.0)	
**Diabetes mellitus within 1 year before index date**	3,152 (4.4)	295 (4.1)	0.266
**Gingival and periodontal diseases** (ICD-9-CM: 523)	23,211 (32.2)	2,897 (40.2)	<0.001
**Acute or chronic periodontitis** (ICD-9-CM: 523.3–4)	15,193 (21.1)	1,933 (26.8)	<0.001
**Chronic periodontitis** (ICD-9-CM: 523.4)	3,014 (4.2)	389 (5.4)	<0.001
**Periodontitis** (ICD-9-CM: 523.3–5)	19,899 (27.6)	2,527 (35.1)	<0.001
**Time gap between the last periodontitis-related visit and the index date, years**	2.5±2.0	2.4±2.1	0.067
< 3 months	1,459 (2.0)	299 (4.2)	<0.001
3 months-6 months	1,557 (2.2)	207 (2.9)	
6 months-1 year	2,680 (3.7)	335 (4.7)	
1–3 years	7,458 (10.4)	846 (11.7)	
>3 years	6,745 (9.4)	840 (11.7)	
**Number of periodontitis-related dental visits**	4.9 ± 4.5	5.6 ± 5.5	<0.001
≤ 2	6,626 (9.2)	710 (9.9)	<0.001
3–4	5,346 (7.4)	658 (9.1)	
4–6	3,313 (4.6)	473 (6.6)	
>6	4,614 (6.4)	6,866 (9.5)	
**Cost of periodontitis-related visits, US$**	141±146	161±159	<0.001
Q1	5,084 (7.1)	548 (7.6)	<0.001
Q2	5,017 (7.0)	548 (8.1)	
Q3	4,955 (6.9)	674 (9.4)	
Q4	4,843 (6.7)	721 (10.0)	
**Number of dental visits without a diagnosis of any periodontal disease (ICD-9-CM: 523)**	8.4±7.7	10.7±9.8	<0.001
≤3	5,895 (8.2)	571 (7.9)	<0.001
4–7	5,315 (7.4)	591 (8.2)	
8–12	4,314 (6.0)	566 (7.9)	
>12	4,375 (6.1)	799 (11.1)	

Results are shown as number (%) unless specified otherwise.

Abbreviations: SLE, systemic lupus erythematosus; SD, standard deviation; OR, odds ratio; CI, confidence interval; ICD-9-CM, International Classification of Diseases, Ninth Revision, Clinical Modification; US$, United States dollars; Q, quartile.

As shown in Table 2, a higher risk of SLE was significantly associated with a history of PD (OR, 1.21; 95% CI, 1.14–1.28; p-value, <0.001), but not DM (OR, 0.92; 95% CI, 0.81–1.05; p-value, 0.199) using multivariable conditional logistic regression analysis.

**Table 2:** Crude and adjusted OR with 95% CI for correlation between variables and the risk of systemic lupus erythematosus using conditional logistic regression analyses.

Variable	OR (95% CI)	P-value
Periodontitis (ICD-9-CM: 523.3–5)	1.21 (1.14–1.28)	<0.001
Diabetes mellitus	0.92 (0.81–1.05)	0.199

Matched variables include age, sex and year of the index date. Adjusted variable includes periodontitis, diabetes mellitus requiring anti-diabetic drugs and number of non-PD related dental visits before the index date.

Abbreviations: OR, odds ratio; CI, confidence interval; ICD-9-CM, International Classification of Diseases, Ninth Revision, Clinical Modification.

Table 3 revealed a consistently significant association between SLE risk and PD exposure using various definitions of PD although this association attenuated mildly using ICD-9-CM code 523.4 as the PD definition.

**Table 3:** Sensitivity analyses for association between systemic lupus erythematous risk and a history of periodontitis based on various definitions shown as adjusted ORs with 95% CIs.

Definition of periodontitis	OR (95% CI)	P-value
Periodontitis (ICD-9-CM: 523.3–5)	1.21 (1.14–1.28)	<0.001
Chronic periodontitis (ICD-9-CM: 523.4)	1.13 (1.01–1.26)	0.033
Acute or chronic periodontitis (ICD-9-CM: 523.3–4)	1.15 (1.08–1.22)	<0.001
Gingival and periodontal diseases (ICD-9-CM: 523)	1.20 (1.13–1.26)	<0.001

Analyses were conducted using the conditional logistic regression model. Matched variables include age, sex and year of the index date. Adjusted variable includes diabetes mellitus requiring anti-diabetic drugs and number of non-PD related dental visits before the index date.

Abbreviations: OR, odds ratio; CI, confidence interval; ICD-9-CM, International Classification of Diseases, Ninth Revision, Clinical Modification.

As shown in Table 4, the correlation between PD and SLE risk was strongest when the interval between the index date and the last PD-related visit date was < 3 months (OR, 1.83; 95% CI, 1.61–2.09), followed by an interval of 3–6 months (OR, 1.23; 95% CI, 1.05–1.43). SLE risk was also higher in patients with more PD-related visits and a higher cumulative cost of their PD-related visits. The above findings suggested a time- and dose-dependent correlation.

**Table 4:** Adjusted OR with 95% CI for association between SLE risk and history of periodontitis using conditional logistic regression analyses.

Variable	OR (95% CI)	P-value
**Time gap between the last periodontitis-related visit and the index date**		
No periodontitis	1.00 (reference)	
< 3 months	1.83 (1.61–2.09)	<0.001
3 months-6 months	1.23 (1.05–1.43)	0.008
6 months-1 year	1.16 (1.03–1.31)	0.014
1 year-3 years	1.08 (0.99–1.17)	0.069
>3 years	1.23 (1.13–1.33)	<0.001
**Number of periodontitis-related dental visits**		
No periodontitis	1.00 (reference)	
≤ 2	1.14 (1.04–1.24)	0.003
3–4	1.20 (1.10–1.32)	<0.001
4–6	1.32 (1.19–1.46)	<0.001
>6	1.25 (1.13–1.37)	<0.001
**Cost of periodontitis-related visits**		
No periodontitis	1.00 (reference)	
Q1	1.13 (1.03–1.24)	0.011
Q2	1.17 (1.06–1.28)	0.001
Q3	1.27 (1.16–1.39)	<0.001
Q4	1.28 (1.17–1.41)	<0.001

Matched variables include age, sex and year of index date. Adjusted variable includes diabetes mellitus requiring anti-diabetic drugs and number of non-PD related dental visits before the index date.

Abbreviations: OR, odds ratio; CI, confidence interval; US$, United States dollars; Q, quartile.

Subgroup analyses according to age, sex, and DM for the association between SLE risk and PD exposure were revealed in Table 5. The association between PD and SLE risk remained statistically significant among all subgroups except in DM patients using any PD definition, and in male patients using ICD-9-CM: 523.4 or ICD-9-CM: 523.3–4 as the PD definition. Age, sex and DM did not have modification effect on the association between PD exposure and SLE risk (p for interaction all >0.05).

**Table 5:** Multivariable analyses for the association between periodontitis and the risk of systemic lupus erythematosus stratified by age, gender and diabetes mellitus (DM).

	Periodontitis (ICD-9-CM: 523.3–5)			Chronic periodontitis (ICD-9-CM: 523.4)			Acute or chronic periodontitis (ICD-9-CM: 523.3–4)			Gingival and periodontal diseases (ICD-9-CM: 523)		
	OR (95% CI)	P-value	P for interaction	OR (95% CI)	P-value	P for interaction	OR (95% CI)	P-value	P for interaction	OR (95% CI)	P-value	P for interaction
**Age group**			0.903			0.428			0.787			0.940
<50 years	1.21 (1.13–1.29)	<0.001		1.08 (0.92–1.27)	0.339		1.16 (1.08–1.25)	<0.001		1.20 (1.13–1.29)	<0.001	
≥50 years	1.20 (1.08–1.33)	<0.001		1.17 (1.00–1.37)	0.048		1.12 (1.01–1.25)	0.037		1.18 (1.06–1.30)	0.002	
**Sex**			0.604			0.675			0.127			0.306
Female	1.20 (1.13–1.27)	<0.001		1.13 (1.00–1.28)	0.049		1.16 (1.09–1.23)	<0.001		1.19 (1.12–1.27)	<0.001	
Male	1.27 (1.09–1.47)	0.002		1.13 (0.86–1.48)	0.382		1.12 (0.95–1.32)	0.172		1.21 (1.05–1.41)	0.011	
**DM**			0.475			0.463			0.729			0.335
No	1.21 (1.14–1.28)	<0.001		1.14 (1.01–1.28)	0.028		1.15 (1.08–1.22)	<0.001		1.20 (1.13–1.27)	<0.001	
Yes	1.18 (0.87–1.61)	0.282		1.04 (0.66–1.64)	0.866		1.21 (0.89–1.66)	0.229		1.16 (0.85–1.58)	0.343	

Abbreviations: ICD-9-CM, International Classification of Diseases, Ninth revision, Clinical Modification; OR, odds ratio; CI, confidence interval; DM, diabetes mellitus.

## Discussion

To our understanding, this study is the first nationwide, population-based, case-control study to examine the association between PD exposure and the risk of SLE. Sensitivity analyses showed the results were not sensitive to varying definitions of PD, while the coding of PD may introduce a misclassification bias. However, considering that it is impossible to calculate the exact misclassification rate, and given the assumption that the misclassification rate for PD was the same for each group in our study, the direction of such nondifferential misclassification bias was always towards the null [29].

This study also revealed both time- and dose-dependent relationship between PD exposure and incident SLE. In this study, we assumed that in patients with a history of PD, its severity correlates with the cumulative number of PD-related visits and the cumulative cost of these visits before the index date.

Of note, we found that the magnitude of the association between PD and SLE risk peaked when the lag time of the last PD-related visit was less than three months (OR, 1.83; 95% CI, 1.61–2.09). However, we cannot rule out the possibility that if the onset of SLE was insidious, SLE might have developed before the last PD-related visit when the lag time was less than three months. Given that a higher prevalence of PD in SLE patients had been reported in previous studies [17, 18], reverse causality may also explain this finding.

The onset of SLE most typically occurs in the childbearing years, after menarche and before menopause. Hormonal contributions to immune system activation (particular estrogen), are likely to explain the female predominance and peak incidence during the reproductive years of age. However, the association between PD exposure and SLE risk did not differ among subgroups stratified according to age or sex.

There are some possible explanations for the association between PD and SLE risk. First, the stimulation of the expression of both TLR-2 and TLR-4 in SLE patients may partially be influenced by PD, which due to the effect of microorganisms involved in the chronic inflammatory process related to their disease process, stimulates the activation mechanisms of autoimmunity related to SLE [30]. Second, in PD patients, over-reactivation of B cells to the antigenic burden, present at the sites of periodontal compromise, would result in polyclonal activation of B cells [31]. Third, the prevalence of patients who are positive for anticardiolipin antibodies in both chronic PD and generalized aggressive PD, was higher than those in healthy controls [32]. Wang et al. showed that active SLE patients who had intraoral habitats of either *Porphyromonas gingivalis* (*P*. *gingivalis*) or *P*. *gingivalis* together with *Treponema denticola* exhibited higher anti-cardiolipin and anti-β2-glycoprotein I antibodies than those without these bacteria [21]. In summary, periodontopathic bacteria infection may interact with the innate and adaptive immune system in several ways, for instance, molecular mimicry, and could eventually lead to abnormal autoantibody production.

Treatment of PD reduces the risk of vascular injuries, and thus decreases the counts of both CD34+ and CD4+ cell types, which are involved in vascular inflammation and repair [33]. Periodontal treatment also has the beneficial effect of controlling disease activity in SLE patients under immunosuppressive therapy [22]. However, to date, there was no evidence showing that aggressive treatment of PD can prevent SLE development in genetically susceptible individuals.

This study had some limitations. First, because smoking is a major risk factor for PD [34] as well as SLE [35, 36], the weak association between PD and SLE might be introduced by unmeasured smoking status. Second, the accuracy of diagnoses based on administrative data is an issue of concern. Although the BNHI routinely samples patient charts randomly to cross-check the quality of claims from all medical institutions, we cannot wholly avoid any bias due to miscoding or misclassification. However, the nondifferential misclassification bias introduced by the diagnosis of PD can only underestimate the strength of the relationship between PD and SLE risk. Also, the accuracy and validity of SLE diagnosis remain less of a concern because the BNHI selected at least two qualified and experienced rheumatologists to validate SLE diagnosis by reviewing patients’ medical charts, and conducting a lupus laboratory test before issuing a catastrophic illness certificate. Third, though we used a medical record to identify the date of SLE diagnosis, SLE may have developed before the diagnosis date. Fourth, a case-control study is limited by its weaker power of causal inference, when compared with a cohort study. Finally, an administrative database cannot provide the needed clinical data to test the association between PD and lupus related auto-antibodies; furthermore, such a database lacks the clinical information required for assessing precise PD severity. However, it is reasonable to hypothesize that PD severity positively correlated with more PD-related visits, along with higher costs of medical care. With this information, our study showed a dose-dependent relationship between PD and SLE risk.

### Conclusion

This nationwide, population-based, case-control study demonstrated a significant association between a history of PD and SLE risk. Further basic and clinical studies are warranted to elucidate whether PD may trigger the development of SLE.

## Supporting information

S1 Data Data of the SLE group and the non-SLE group. (SAV)
